# BERT-Based Natural Language Processing of Drug Labeling Documents: A Case Study for Classifying Drug-Induced Liver Injury Risk

**DOI:** 10.3389/frai.2021.729834

**Published:** 2021-12-06

**Authors:** Yue Wu, Zhichao Liu, Leihong Wu, Minjun Chen, Weida Tong

**Affiliations:** Division of Bioinformatics and Biostatistics, National Center for Toxicological Research, United States Food and Drug Administration, Jefferson, AR, United States

**Keywords:** regulatory science, drug labeling, natural language processing, BERT, drug induced liver injury, United States Food and Drug Administration, European medicines agency, named entity recognition

## Abstract

**Background & Aims:** The United States Food and Drug Administration (FDA) regulates a broad range of consumer products, which account for about 25% of the United States market. The FDA regulatory activities often involve producing and reading of a large number of documents, which is time consuming and labor intensive. To support regulatory science at FDA, we evaluated artificial intelligence (AI)-based natural language processing (NLP) of regulatory documents for text classification and compared deep learning-based models with a conventional keywords-based model.

**Methods:** FDA drug labeling documents were used as a representative regulatory data source to classify drug-induced liver injury (DILI) risk by employing the state-of-the-art language model BERT. The resulting NLP-DILI classification model was statistically validated with both internal and external validation procedures and applied to the labeling data from the European Medicines Agency (EMA) for cross-agency application.

**Results:** The NLP-DILI model developed using FDA labeling documents and evaluated by cross-validations in this study showed remarkable performance in DILI classification with a recall of 1 and a precision of 0.78. When cross-agency data were used to validate the model, the performance remained comparable, demonstrating that the model was portable across agencies. Results also suggested that the model was able to capture the semantic meanings of sentences in drug labeling.

**Conclusion:** Deep learning-based NLP models performed well in DILI classification of drug labeling documents and learned the meanings of complex text in drug labeling. This proof-of-concept work demonstrated that using AI technologies to assist regulatory activities is a promising approach to modernize and advance regulatory science.

## Introduction

The United States FDA regulates consumer products including foods, medications and tobacco, which account for about 25% of the United States market (US Food and Drug Administration, 2011a). The core responsibility of FDA is to ensure safe and effective products, while at the same time promote innovation to produce products of better quality (US Food and Drug Administration, 2010). Therefore, FDA must be equipped with the best available tools and methods to facilitate pre-market evaluation and post-market surveillance, which requires a strong field of regulatory science to develop standards and approaches that assess FDA-regulated products with reliable efficiency and consistency (US Food and Drug Administration, 2011a; Hamburg, 2011).

Currently, science and technology are rapidly evolving in the field of healthcare, introducing more complexity to the development and manufacture of new drugs, biologics and medical devices. Artificial intelligence (AI), especially, is a fast-growing area and has shown great potential in addressing the unmet medical and public health needs (Yu et al., 2018; Basile et al., 2019; Chan et al., 2019). A long-lasting challenge for FDA is to efficiently retrieve needed information from a huge number of documents received and regularly generated, such as approval documents, guidance, policies and meeting minutes. A significant amount of time must be spent on manually reading and searching information of interest, besides product evaluation and decision making. AI-based natural language processing (NLP) is a promising approach of speeding up this time-consuming and labor-intensive process.

In this study, we applied AI-based NLP to classify drug labeling documents as a proof-of-concept to demonstrate the utility of AI for regulatory applications. Drug labeling provides comprehensive summaries of medications as a reference for healthcare professionals in making prescribing decisions (Watson and Barash, 2009; McMahon and Preskorn, 2014). It is also an essential resource for FDA reviewers during drug evaluations, and the research community for pharmacovigilance and drug repositioning (Chen et al., 2011, 2016; Hoffman et al., 2016; Fang et al., 2020). There are over 130,000 drug labeling documents in the repository, of which 47,000 are labeling for prescription drugs and biologics (Fang et al., 2020). This represents large amounts of regulatory text data, making manually assessing all drug labeling documents prohibitory, if not impossible. Here, we developed an AI-based approach to classify drug-induced liver injury (DILI) risk indicated in drug labeling documents, which serves as a proxy to test the applicability of AI in facilitating text classification from regulatory documents.

Adverse drug reactions (ADRs) such as DILI are described in three sections, “Adverse Reactions”, “Warnings and Precautions” and “Boxed Warning”, in FDA drug labeling documents (US Food and Drug Administration, 2006; US Food and Drug Administration, 2011b). The “Warnings and Precautions” section contains the most comprehensive and complicated descriptions not limited to ADRs, but also includes other related aspects such as warnings to patients for signs and symptoms, clinical/laboratory monitoring plans and contraindications, for which sentences containing DILI-related terms do not necessarily suggest attributable DILI events (US Food and Drug Administration, 2011b). In contrast, the “Boxed Warning” section, specific to FDA labeling, contains concise highlights of the most serious ADRs from the “Warnings and Precautions” section (US Food and Drug Administration, 2011b), while the “Adverse Reactions” section more or less lists all possible ADRs (US Food and Drug Administration, 2006). The current manual classification approach largely relies on the use of pre-defined DILI terms to determine whether sentences in the three labeling sections indicate DILI (Chen et al., 2011; 2016). Considering that the terms used in the drug labeling are not well normalized to the international standards such as Medical Dictionary for Regulatory Activities (MedDRA) and Systematized Nomenclature of Medicine (SNOMED) and the complexity of language used for describing ADRs, interpretation and judgement by experts with relevant knowledge and experience are necessary. We used an AI-based approach to address these issues in the current study, as language models can capture the semantic meanings of sentences in free text rather than simple string matching (Radford et al., 2018). Specifically, the state-of-the-art language model, Bidirectional Encoder Representations from Transformers (BERT) (Devlin et al., 2019), was trained for binary DILI classification of FDA-approved drug labeling documents and was externally validated using EMA-approved drug labeling documents. The deep learning-based model, hybrid deep learning-based model and keywords-based model developed in this study were compared for DILI risk classification on drug labeling documents.

## Materials and Methods

### Data Sources for Drug Labeling

FDA drug labeling documents were retrieved from DailyMed (www.dailymed.nlm.nih.gov), a public database that contains up-to-date drug labeling approved by the FDA. Meanwhile, since the EMA issues standardized drug labeling for drugs approved through a centralized procedure, we used UK-marketed drugs as representatives of drugs authorized in Europe (European Medicines Agency, 2009). EMA drug labeling documents were collected from the EMC (www.medicines.org.uk), which maintains the EMA-approved drug labeling for drugs licensed in the United Kingdom.

### Drug Selection Criteria

We selected prescription drugs based on three criteria, i) with a single active ingredient, ii) either oral or injection use, and iii) in the categories of NDA, ANDA or BLA, by querying the FDALabel database (https://nctr-crs.fda.gov/fdalabel/ui/search) which maintains over 130,000 drug labeling documents containing critical information pertinent to the safe and effective use of medications (Fang et al., 2020). Over-the-counter drugs were removed because of their different labeling format and requirements compared to prescription drugs. The DILIrank dataset provides the DILI risk annotation for 1,036 drugs marketed in the United States as of 2010 (Chen et al., 2016). We retrieved the most recent drug labeling documents for the queried 750 representative prescription drugs from the DILIrank dataset. Among these drugs, 540 were also licensed in the United Kingdom market. The corresponding EMA drug labeling documents were collected and assessed for DILI risk using the same classification schema described in previously studies (Chen et al., 2011).

### Datasets

**FIGURE 1:**
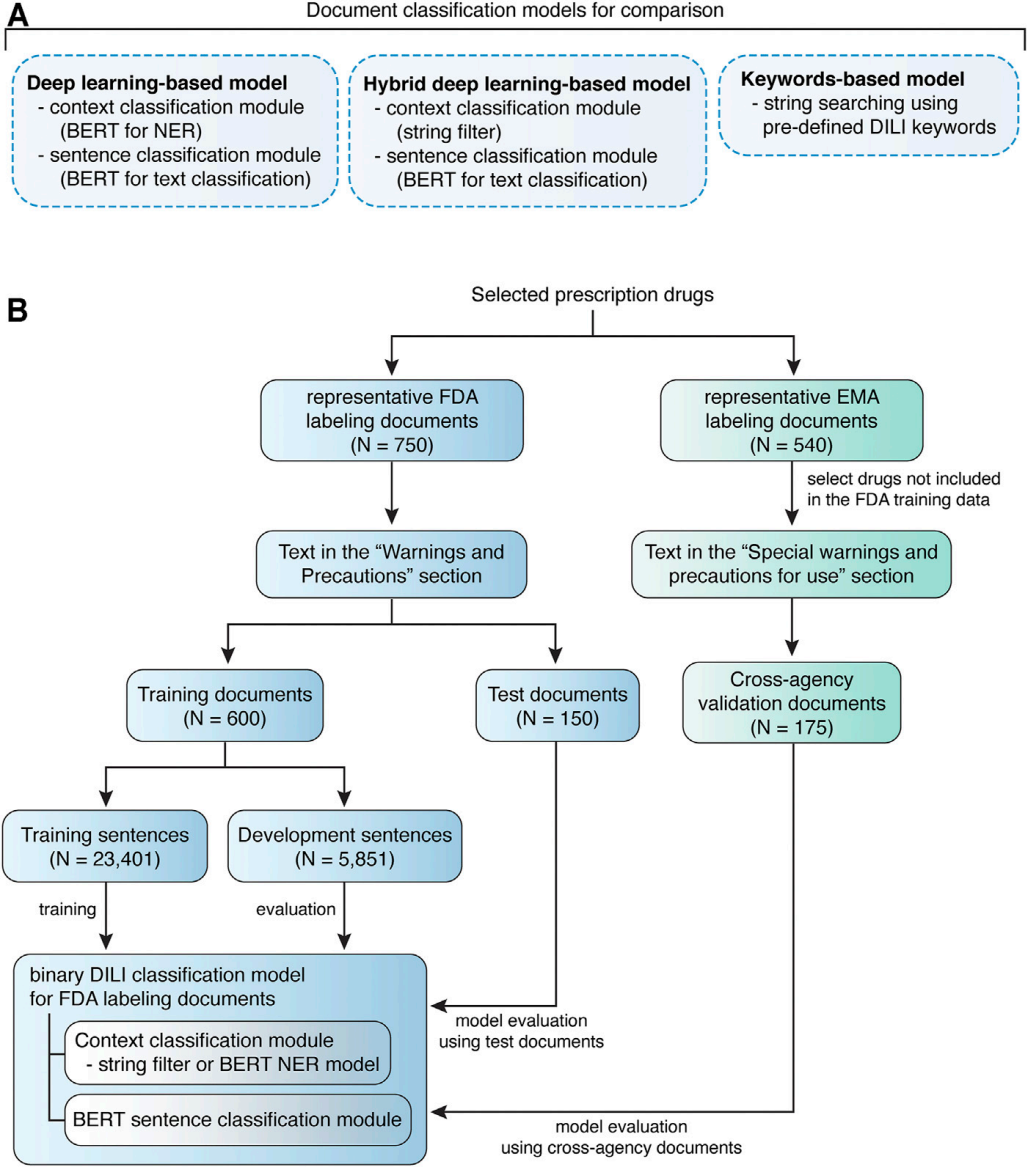
Quorum flowchart describes the study design. **(A)** Drug labeling document classification models developed and compared in this study. **(B)** The study design of model training and evaluation using FDA labeling documents and model validation using EMA labeling documents.

**FIGURE 2:**
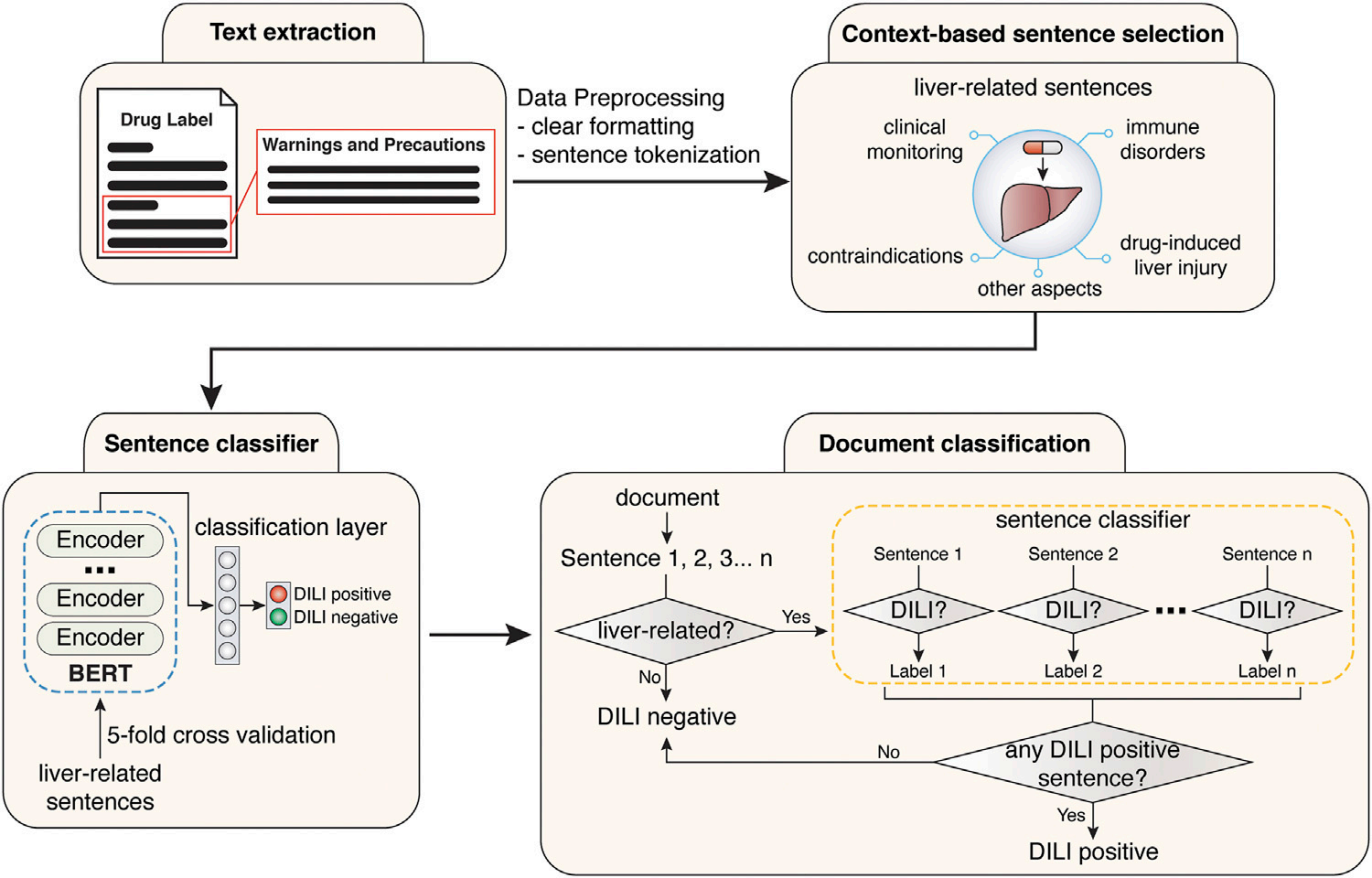
Workflow for the training of sentence classification module and the development of final document classification model.

We focused our analysis on the “Warnings and Precautions” section of FDA labeling documents, as the language for ADR descriptions in this section has the highest complexity compared with the other two sections (US Food and Drug Administration, 2011b). The corresponding section in the EMA labeling documents is the “Special warnings and precautions for use” section (European Medicines Agency, 2009). Texts were extracted from either the “Warnings and Precautions” section (FDA) or the “Special warnings and precautions for use” section (EMA), followed by formatting clearing and sentence tokenization (Figures 1B, 2).

For model training on FDA labeling documents, the representative documents (N = 750) were stratified split into 80% training document dataset (N = 600) and 20% test document dataset (N = 150). Unique sentences (N = 29,252) were extracted from the training document dataset, among which DILI-positive (N = 540) or DILI-negative sentences (N = 28,712) were determined independently by two experts. All disagreements were resolved by discussion. To generate data with more balanced class labels, intermediate datasets were created to facilitate filtering of context prior to sentence classification, via Named Entity Recognition (NER). The unique sentences (*N* = 29,252) from training documents were annotated using the Inside-Outside-Beginning (IOB) style. The annotated sentences were randomly split into 80% training sentence dataset (N = 23,041) and 20% development sentence dataset (N = 5,851) for NER model training (Figure 1B). Sentences with tokens related to a liver context, 540 DILI-positive and 1,313 DILI-negative, were selected as liver-related sentences. To simplify the comparison between models, human validated liver-related sentences from the annotated sentences (*N* = 29,252) were used for developing the sentence classification module. Test document dataset was used to evaluated developed models, and cross-agency data, i.e., EMA labeling documents for drugs not included in the FDA training data, was used for external validation (Figure 1B).

Examples are given here to illustrate the datasets created for model training. Dataset for context classification included liver-related sentences such as “Hepatic toxicity including hepatic failure resulting in transplantation or death have been reported” and “Rozerem should not be used by patients with severe hepatic impairment” and sentences irrelevant to liver including “Treat all infections due to Group A beta-hemolytic streptococci for at least 10 days”. The first two liver-related sentences were used for developing sentence classification models. The first sentence was considered as DILI-positive, while the second sentence is for contraindication information and thus considered as DILI-negative.

To further examine the portability of BERT-based models across agencies, we also developed models using EMA labeling documents as training data and validated the models using FDA labeling documents (Supplementary Figure 1). EMA labeling documents (N = 540) were stratified split into 80% training document dataset (N = 431) and 20% test document dataset (N = 109). Unique sentences (N = 14,915) were extracted from the training document dataset, including 232 DILI-positive and 14,683 DILI-negative sentences. Similarly, intermediate datasets were created to facilitate filtering of context prior to sentence classification, via NER. The unique sentences (*N* = 29,252) from training documents were annotated using the IOB style, and randomly split into 80% training sentence dataset (*N* = 11,931) and 20% development sentence dataset (*N* = 2,984) for NER model training (Supplementary Figure 1). Sentences with tokens related to a liver context, 232 DILI-positive and 927 DILI-negative, were selected as liver-related sentences. Human validated liver-related sentences from the annotated sentences (*N* = 14,915) were used for developing the sentence classification module. EMA test document dataset was used to evaluated developed models, and FDA labeling documents for drugs not included in the EMA training data, was used for external validation.

### Models for Document Classification

In this study, deep learning-based (BERT for DILI classification), hybrid deep learning-based and keywords-based models were developed for classifying drug labeling documents based on whether they contain any sentence suggesting DILI risk (Figure 1A).

The deep learning-based and hybrid deep learning-based document classification models consisted of two working modules, a context classification module and a BERT sentence classification module (Figures 1A, 2). These two models shared the same BERT sentence classification module but differed in the context classification module. For each input document, each sentence was passed into the two working modules sequentially (Figure 2). The first step was to determine whether the current sentence was related to the liver topic at the context classification module. If not, this sentence was DILI-negative. If yes, this sentence was then passed to the BERT sentence classification module to determine whether it was DILI-positive or DILI-negative. After evaluating all the sentences in the input document, an array of predicted sentence labels was generated. If any DILI-positive sentences were found in the input document, the document was considered DILI-positive, otherwise as DILI-negative.

A keywords-based document classification model was also developed as a comparison to the deep learning-based and hybrid deep learning-based models (Figure 1A). Keywords for detecting DILI risk in the drug labeling were collected from three previous studies (Chen et al., 2011; Demner-Fushman et al., 2018; Suzuki et al., 2015) (Supplementary Table 1). Chen et al. summarized a list of DILI keywords for text-mining (via human reading) in the drug labeling, while Suzuki et al. selected a list of MedDRA PT terms for hepatocellular and cholestatic liver injury for text-mining in the WHO VigiBase™. These two lists covered most of the DILI terms, but the keywords commonly had multiple imperfect matches in the drug labeling documents. Thus, these keywords could not be used directly for computerized text-mining in the drug labeling documents. Demner-Fushman et al. normalized the ADR terms in 200 drug labeling documents to MedDRA Preferred Terms (PTs). By using the matching data in the Demner-Fushman et al. study, we generated a keyword list that covers DILI (Chen et al., 2011), liver injury (Suzuki et al., 2015) and hepatic ADRs (Demner-Fushman et al., 2018) terms used in drug labeling. The FDA and EMA test document sets were used to evaluate the performance of keywords-based document classification.

### Development of the Context Classification Modules

Two types of context classification modules were created in this study. The first one is a string pattern matching-based context filter. The other one is an NER-based context classification model.

For the hybrid deep learning-based model, general string patterns were used to match sentences with any possible relation to liver, including indications, contraindications, ADRs, clinical monitoring, immune disorders, etc. (Supplementary Table 2). Most DILI-negative sentences irrelevant to liver were filtered out by applying such pre-defined context, yielding relatively balanced sentence datasets without losing any DILI-positive sentences (Table 1).

**TABLE 1:** Sentence count with or without pre-defined liver-related context.

	Without pre-defined context		In context of liver (string-filter)		In context of liver (BERT for NER)	
	FDA	EMA	FDA	EMA	FDA	EMA
DILI positive sentences	540	232	540	232	540	232
DILI negative sentences	28,712	14,915	961	764	1,313	927

Meanwhile, a BERT-based NER model was developed as the context classification module in the deep learning-based model. The NER model was developed by using training sentence dataset and evaluated on development sentence dataset at each epoch of training. The hyperparameters used for model training are listed in Supplementary Table 3. This BERT-based context classification module was then evaluated by performing context classification on sentences extracted from test documents and cross-agency validation documents.

### Development of the BERT-Based Sentence Classification Module

The liver-related sentences selected from training sentence dataset were used for developing a BERT (base, uncased) model for binary DILI classification as the sentence classification module, while the liver-related sentences selected from development sentence dataset were used to evaluate the performance of the BERT-based sentence classification module. The hyperparameters used for model training are listed in Supplementary Table 4. The sentence classification module was evaluated using shuffled five-fold cross-validations on the liver-related sentences for 100 times (Supplementary Figure 2). In comparison to developing a context-dependent sentence classification model, we also trained a sentence classification model using imbalance sentence datasets extracted from training documents. To address the dataset imbalance issue, we applied an oversampling method, i.e., randomly sampling based on class weights.

Permutation analysis was conducted to determine whether the models developed in this study perform at chance (Chen et al., 2013). Permutated datasets were generated by 100 times of resampling the liver-related training and test sentence datasets with randomly shuffled DILI classification labels (positive or negative). The performance of the resulting 100 models was compared with that from 100 repetitions of cross-validations with random sampling (Supplementary Figure 2). A two-sided *t*-test was used determine the statistical significance of the difference between the accuracy scores obtained from permutated data and original data.

Shapley Additive Explanations (SHAP) values (Lundberg and Lee, 2017) were used to quantify the contribution of each token to the prediction made by the model. Higher feature values (red) push the model prediction towards DILI-positive, while lower features (blue) values push the model prediction towards DILI-negative.

### Implementation

The embedding layer and 12-layer encoder from BERT were adopted and connected with a dense layer for token or sentence classification. The deep learning-based model combines NER (token classification) and sentence classification modules. A document is broken down into sentences *s*_*1*_ *, s*_*2*_…*s*_*i*_. All sentences are passed into the NER module, where tokens [*t*_*11*_ *, t*_*12*_…*t*_*1j*_], [*t*_*21*_ *, t*_*22*_…*t*_*2j*_]…[*t*_*i1*_ *, t*_*i2*_…*t*_*ij*_] are classified. If none of the tokens is associated with “Liver” (with (*argmax(t*_*i1*_ *)* = *y*) | (*argmax(t*_*i2*_ *)* = *y*) | … | (*argmax(t*_*ij*_ *)* = *y*) being False for any sentence *s*_*i*_ in a given document, where y equals the value of “Liver” tag.), then document label is returned as 0 (DILI negative). Otherwise, all selected liver related sentences are passed into sentence classification module. Document label is returned as 0 if none of the liver-related sentences is DILI positive ((*∑*_*i*_ *argmax(s*_*i*_ *)* = 0), else returned as 1 (DILI negative).

### Evaluation Metrics

The NER-based context classification was evaluated at two levels. Recall, precision, and f1-score were reported at token level. Context classification at sentence level was evaluated by recall and precision. The BERT-based binary sentence classification was evaluated using accuracy, recall and precision. The test documents were used to assess the performance of the deep learning-based and hybrid deep learning-based models on document classification. Matthews correlation coefficient (MCC), recall and precision were used to evaluate the quality of binary DILI classification predicted by the models.

## Results

### Development of the Deep Learning-Based Model for DILI Classification of Labeling Documents

**FIGURE 3:**
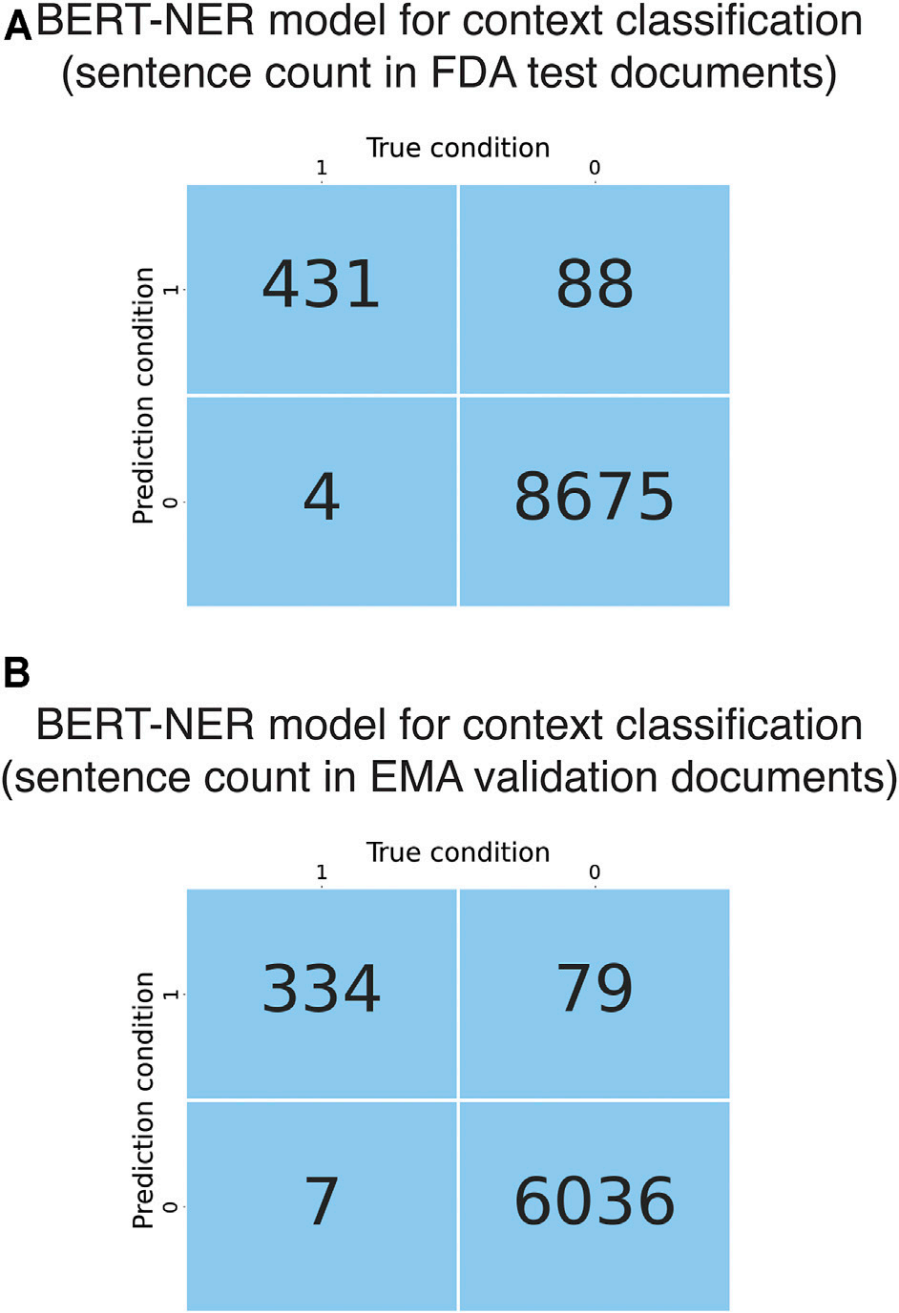
Evaluation and validation of the BERT NER models for context classification. **(A)** Confusion matrix obtained from evaluation of the BERT-based context classification module using the FDA test documents. **(B)** Confusion matrix obtained from evaluation of the BERT-based context classification module using the EMA validation documents.

The developed deep learning-based model had a BERT-based NER model as the context classification module and a BERT-based sentence classification module (Figure 1A). FDA test documents were used to evaluate the performance of the NER-based context classification module in selecting liver-related sentences. At token level, the context classification module showed excellent performance in recognizing liver-related words, with an F1 score of 0.98 ± 0.003, recall of 0.99 ± 0.002 and precision of 0.98 ± 0.008. When evaluated at sentence level, it had great sensitivity (0.99) as it was able to extract 431 of 435 liver-related sentences from the test documents (Figure 3A). The precision was 0.83 (0.83 ± 0.001 from cross-validations) due to that 88 false positives were generated. Considering the large number of non-liver sentences (N = 8,763) in the test documents, the context classification module performed well in predicting non-liver sentences as the false positive rate was 1%. Further, the context classification module was externally validated using EMA test documents. It detected 334 of 341 liver-related sentences while 79 false positives were predicted from 6,115 non-liver sentences, which was comparable to the results obtained using FDA test documents (Figure 3B).

The BERT-based sentence classification module is the same from the hybrid deep learning-based model, which was developed using liver-related sentences. This module showed an accuracy of 0.81 ± 0.02, recall of 0.82 ± 0.03 and precision of 0.82 ± 0.02. To confirm that the sentence classification module did not perform at chance, we conducted permutation tests. The sentence classification models trained on the permutated FDA training sentences exhibited a great decrease in average accuracy score, as compared to that obtained from cross-validations (0.56 versus 0.81, *p* < 0.0001) (Supplementary Figure 2). These results suggested that the observed accuracy scores of the sentence classification models were unlikely to be obtained by chance.

**FIGURE 4:**
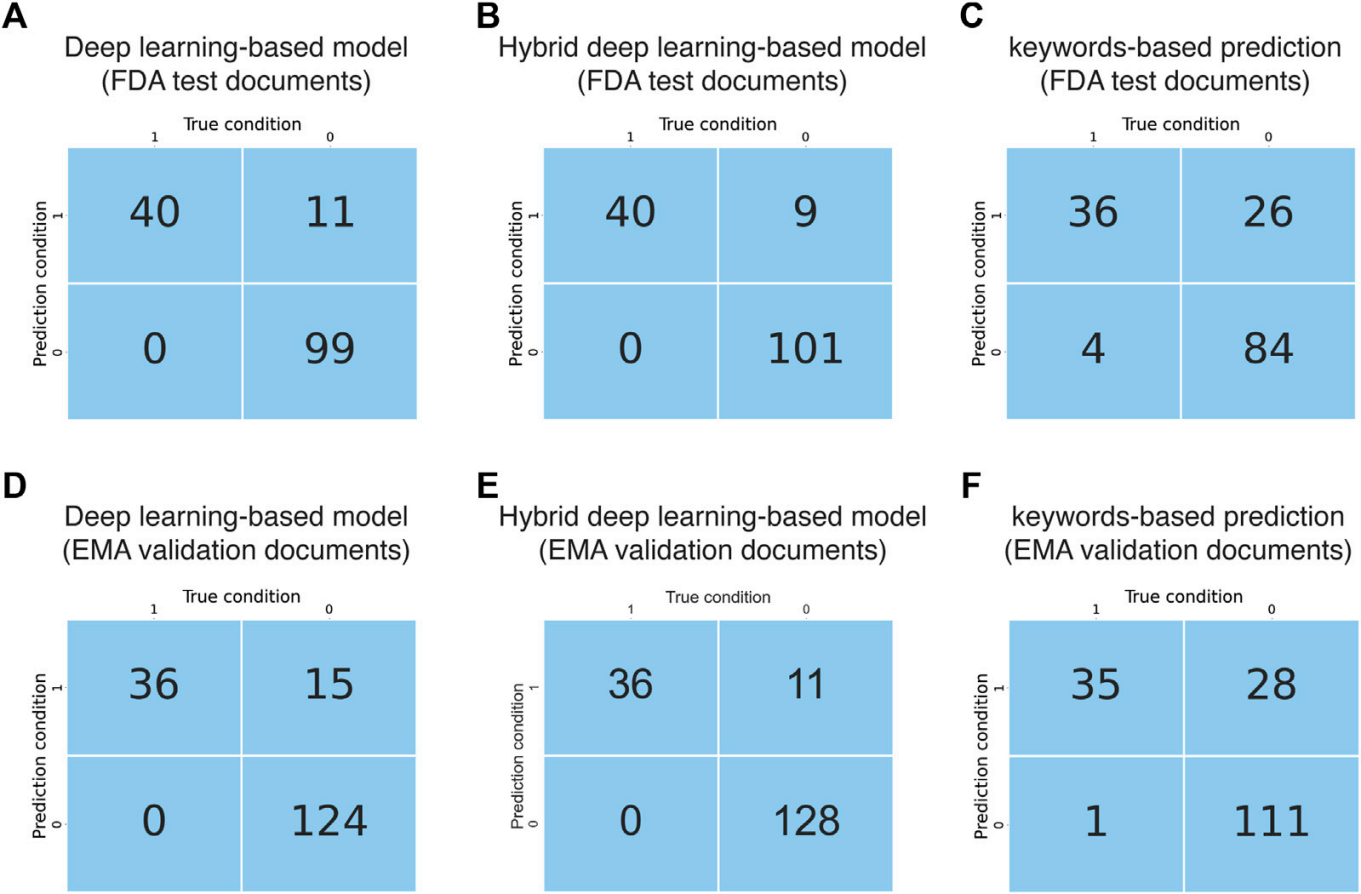
Evaluation and validation of the document classification models. **(A)** Confusion matrix obtained from evaluation of the AI model using FDA test documents. **(B)** Confusion matrix obtained from evaluation of the hybrid deep learning-based model using FDA test documents. **(C)** Confusion matrix obtained from evaluation of the keywords-based model using FDA test documents. **(D)** Confusion matrix obtained from evaluation of the AI model using EMA validation documents. **(E)** Confusion matrix obtained from evaluation of the hybrid deep learning-based model using EMA validation documents. **(F)** Confusion matrix obtained from evaluation of the keywords-based model using EMA validation documents.

The performance of the deep learning-based model regarding document classification was evaluated using FDA test documents and externally validated using EMA validation documents (Figure 1B). The deep learning-based model also showed excellent performance in DILI prediction on drug labeling documents with an MCC of 0.84 (Table 2). It could detect all 40 of the DILI-positive documents in the FDA test set (Figure 4A and Table 2). Eleven false positives were found from a total of 110 DILI-negative documents, and thus the precision was 0.78. These results were consistent with that from model validation using cross-agency data (EMA validation documents), which had an MCC of 0.79, recall of 1 and precision of 0.71 (Figure 4D and Table 2).

**TABLE 2:** Model evaluation and validation using cross-agency data.

Model evaluation using FDA test documents			
Document classification models	Matthews correlation coefficient	Recall	Precision
Deep learning-based model	0.84	1.00	0.78
Hybrid deep learning-based model	0.87	1.00	0.82
Keywords-based model	0.60	0.90	0.58
Model validation using cross-agency data (EMA test documents)			
Document classification models	Matthews correlation coefficient	Recall	Precision
Deep learning-based model	0.79	1.00	0.71
Hybrid deep learning-based model	0.84	1.00	0.77
Keywords-based model	0.61	0.96	0.55

In comparison with models trained on liver-related sentences, we also developed sentence classification models using all sentences from the training documents, which were extremely imbalanced between DILI-positive and negative labels. We observed decreased recall (0.75 ± 0.08) and precision (0.76 ± 0.04) as compared to models developed using liver-related sentences. When oversampling was conducted by randomly sampling according to class weights, recall was increased to 0.80 ± 0.04 while precision dropped significantly to 0.68 ± 0.04. None of these models outperformed the deep learning-based model with NER-based intermediate module at sentence level. When evaluated at document level, the sentence classification model trained on all sentences predicted more false negative FDA documents (*N* = 4), causing decreased recall (0.90). Interestingly, precision (0.86) was higher than that obtained from the deep learning-based model, as less false positive documents were obtained (*N* = 6). Similarly, decreased recall (0.89) and increased precision (0.82) were observed when EMA documents were used as external validation data. Higher recall is preferred for the investigated topic in this study, i.e., ADR detection in drug labeling documents, because false positive documents are much easier to be detected during the phase of result interpretation or model validation, as compared to false negative documents.

### Development of the Hybrid Deep Learning-Based Model for DILI Classification of Labeling Documents

The developed hybrid deep learning-based model had a string filter-based context classification module followed by a BERT-based sentence classification module (Figure 1A). After context filtering of sentences from the “Warnings and Precautions” section of FDA training documents, 1,501 unique liver-related sentences were collected, of which 540 were DILI-positive while 961 were DILI-negative (Table 1). This sentence dataset was used for training the BERT-based sentence classification module. The developed sentence classification module reached high performance regarding DILI classification with accuracy scores of 0.81 ± 0.02 obtained from 100 repetitions of five-fold cross-validations.

The performance of this hybrid deep learning-based model regarding document classification was evaluated using FDA test documents and externally validated using EMA validation documents (Figures 1B, 2). The hybrid deep learning-based model achieved excellent performance in DILI prediction on drug labeling documents with an MCC of 0.87 (Table 2). It had a high recall of 1, as it could detect all 40 of the DILI-positive documents in the FDA test set (Figure 4B and Table 2). Nine false positives were found which resulted in a precision of 0.82. These results were corroborated with that from model validation using cross-agency data (EMA test documents). The hybrid deep learning-based model had a consistent MCC of 0.84, recall of 1 and precision of 0.77 when predicting on the EMA validation documents (Figure 4E and Table 2).

Interestingly, we observed subtle differences between the deep learning-based and hybrid deep learning-based models in prediction DILI risk. The hybrid deep learning-based model was better at distinguishing liver injury statements in animal studies from human liver injury statements. Also, hepatosplenic T-cell lymphomas due to immunosuppressive treatment could confuse the deep learning-based model rather than the hybrid deep learning-based model. In contrast, the deep learning-based model performed better in detecting term variants/abbreviations, such as SGOT/AST for aspartate aminotransferase and SGPT/ALT for alanine aminotransferase. Although limited in number, the examples from the current data could provide some insight for future research.

### Comparison of the Deep Learning-Based and Hybrid Deep Learning-Based Models With the Keyword-Based Model for DILI Classification of Labeling Documents

As a comparison to the deep learning-based and hybrid deep learning-based models, a keyword matching-based approach was also used to classify the FDA and EMA test documents. The keyword-based classification on FDA test documents showed a significantly lower MCC of 0.60, as compared to that from predictions made by the deep learning-based (0.84) and hybrid deep learning-based (0.87) models (Table 2). It produced a larger number of false positives (N = 26), thus the precision (0.58) was remarkably lower than the deep learning-based (0.78) and hybrid deep learning-based (0.82) models (Figure 4C and Table 2). Most of the false positives produced by keyword-based DILI classification, but not by the deep learning-based and hybrid deep learning-based models, were related to description of contraindications or precautions to special populations (e.g., patients with hepatic impairment) and hypersensitivity reactions (Supplementary Table 5). Also, four false negatives were generated by the keywords-based document classification model, but none by deep learning-based and hybrid deep learning-based models. Corroborated with the DILI classification results obtained from the FDA test documents, the keywords-based DILI classification on the EMA validation documents also showed poor performance in controlling the number of false positives, which generated a low precision of 0.55 (Figure 4F and Table 2). The MCC was calculated to be 0.61.

## Discussion

In this study we used an AI-based NLP approach to classify drug labeling documents according to the DILI risk suggested in the text from the “Warnings and Precautions” section. The motivation of this investigation was to address two questions that are important to both regulatory application and drug safety research, i) whether AI-based NLP tools can be used to classify a drug’s DILI potential specified in the drug labeling documents, and ii) whether an AI-based model developed using FDA labeling documents was portable to the documents in other regulatory agencies with comparable performance. Therefore, we developed BERT-based deep learning models for DILI classification, which were rigorously evaluated in this study.

**FIGURE 5:**
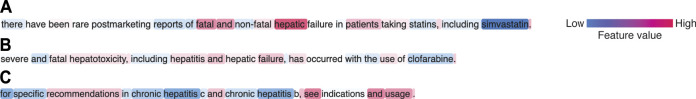
Representative sentences showing contributions of word tokens to model predictions. **(A)** DILI-positive sentence due to fatal hepatic failure. **(B)** DILI-positive sentence due to hepatitis/hepatic failure. **(C)** DILI-negative sentence that provides indication information.

Our results showed that both the deep learning-based model and the hybrid deep learning-based model developed in this study had outstanding performance in predicting DILI risk encoded in the drug labeling documents, regardless of whether FDA labeling documents or EMA labeling documents were used for model training. This suggested that the deep learning-based models could capture the semantic meanings of sentences in the drug labeling documents, considering that the descriptions approved by the two agencies have some degree of difference in terms of language style and format. The contributions of word tokens to model predictions were explored to examine whether the model learned reasonable semantic meanings of the sentences in the drug labeling. SHAP values were used to quantify the contributions of each word token to the prediction made by the model. In the representative DILI-positive sentences (Figures 5A,B), DILI-related words such as “hepatic failure”, “hepatotoxicity” and “hepatitis” showed positive contributions (red) and pushed the model prediction toward DILI-positive. In contrast, the word “hepatitis” did not have positive contributions when it was in the phrases “chronic hepatitis B” and “chronic hepatitis C”. Collectively, these results suggested that the developed NLP models could capture the semantic relationships between words in a given sentence.

Notably, the deep learning-based NLP models developed using FDA labeling documents could also be used by other agencies such as EMA without a notable decrease in performance. Furthermore, we also developed a deep learning-based model and a hybrid deep learning-based model using EMA labeling documents (Supplementary Figure 1). The models trained on the EMA data showed comparable performance when evaluated using EMA test documents and the FDA validation documents (Supplementary Table 6), which confirmed the portability of the deep learning-based NLP models across agencies. This demonstrated a promising potential of using AI technology to facilitate regulatory activities including drug evaluation and pharmacovigilance.

To best resemble our human reading-based approach and allow for an interpretable classification, we chose a sentence classification strategy over directly using whole documents as input. Briefly, we wanted our final model to be able to select liver-related sentences and determine whether they suggest DILI risk. The determination of DILI risk of a document was not based on quantitative measurement of the number of DILI-positive sentences, but rather dependent on detection of at least one DILI-positive sentence. In this regard, the document classification model is sensitive to false positives. Both the FDA and EMA models developed in this study had low false positive rates (6–10%), suggesting that the models performed well in controlling false positives. Furthermore, the sentence classification strategy allowed us to easily track which sentences in a document were the basis for the document classification model to determine DILI potential. It also provided information regarding what type of sentences were ambiguous in DILI risk to the models. From a technical perspective, the current BERT pre-trained model has an input limit of 512 tokens. In order to process lengthy documents such as the “Warnings and Precautions” section containing hundreds to thousands of words, various solutions have been proposed, including i) text truncation and ii) text splitting combined with different pooling methods or Long Short-Term Memory networks (Adhikari et al., 2019a, 2019b; Sun et al., 2020). Such more complex model structures do not fit better the classification criteria for this study and complicate the model interpretation, as compared to a sentence classification-based model structure. Therefore, we used a hierarchical model structure to predict DILI risk on each individual sentence in a given drug labeling document and output a document classification label based on the combined sentence classification results. Moreover, since not all sentences should contribute to the DILI prediction, we used a context filter as a gating mechanism to select liver-related sentence for DILI prediction, which is similar to aspect-based sentiment analysis (Sun et al., 2019; Xu et al., 2019; Choi et al., 2020). The framework for creation of dataset and training of context classification model can be extended to other topics, e.g., cardiotoxicity, drug indication and drug-drug interactions. Outputs from context-classification can also be used for information retrieval pipelines.

Of note, sentence classification models trained on all sentences with skewed distributions did not have dramatically decreased performance than NER-sentence classification combined models. We observed 7 and 6% drop in recall and precision respectively at sentence level, and 10% decrease in recall but 8% increase in precision at document level. However, addition of an NER-based context classification module would be a better approach for the following reasons. First, all the BERT-based models developed in this study were designed to record sentences that were predicted as DILI-positive for human justification. Since the number of sentences suggesting adverse events is far less than that of sentences carrying no information of adverse events, it is much easier to find false positive documents as compared to false negative documents. Also, the false positive sentences collected from users could be used later for model improvement by further training or re-training. Therefore, higher recall is preferred. Second, inclusion of NER-based context classification module enables context-specific sentence classification, which is more flexible, especially in the case of classifying sentences belong to multiple contexts. For example, DILI can be associated with immune-mediated cutaneous ADRs such as Drug Reaction with Eosinophilia and Systemic symptoms, Stevens-Johnson syndrome and toxic epidermal necrolysis (Andrade et al., 2019). Sentences containing information across different contexts could be ambiguous to multiclass sentence classification models for detecting different types of ADRs. If binary sentence classification models were developed for detecting each type of ADRs, large number of negative samples would be used for model training repeatedly, which is not an efficient design. Moreover, NER-based context classification module is versatile and can provide additional functionalities including facilitating information retrieval.

Previous efforts in data mining of drug labeling documents primarily relied on the use of specific ADR terms (Chen et al., 2011; Demner-Fushman et al., 2018; Wu et al., 2019). International standards, MedDRA and SNOMED, have been used for searching ADR terms in drug labeling (Demner-Fushman et al., 2018; Wu et al., 2019). The ADR descriptions in drug labeling often do not follow these standards, which requires human effort in matching ADR terms in drug labeling with standards. Annotation resources have been reported to normalize the terms used in drug labeling (Demner-Fushman et al., 2018). However, providing annotations for such a large repository is not a trivial task. As shown in Supplementary Table 3, many standard terms such as MedDRA PTs have a number of matched terms in drug labeling. For example, there have been at least 31 different terms in FDA labeling for the MedDRA PT “Alanine aminotransferase increased”, and 34 for “Blood bilirubin increased”. New variations in ADR terms are likely to be introduced into drug labeling in the future. Therefore, updating and maintaining such annotations are labor intensive. The deep learning-based model developed in the current study, with BERT-based NER and sentence classification combined, outperformed the keywords-based model by a large margin. Importantly, BERT-based models are not only easy to implement and extend but can also be further improved with better pretrained models in the future.

Furthermore, DILI classification of the labeling documents is a more complicated task than keywords matching. In some cases, a sentence containing hepatic ADR terms does not necessarily suggest DILI. For example, a sentence containing the term hepatitis could indicate antiviral treatment of hepatitis B viruses. It could also be contraindication information specifying that patients with hepatic deficiency due to hepatitis should not take the drug. All these cases are present in the complex descriptions from the “Warnings and Precautions” section. Therefore, human interpretation has been necessary to determine DILI-positive sentences in drug labeling documents (Chen et al., 2011; 2016).

Over the past few years, transformers models have changed the landscape of NLP (Wolf et al., 2020). The BERT model used in this study enables bidirectional text learning by using masks (Devlin et al., 2019). Notably, the multi-headed attention architecture leverages the use of deep neural networks to capture the relationships between words within a sentence and across sentences (Vaswani et al., 2017; Devlin et al., 2019). These two important features allow the BERT model to learn the semantic meanings of a sentence or sentences effectively and efficiently. Thus, we chose BERT as our first attempt to develop AI-based NLP tools, which do not rely on keywords dictionaries but rather learn the meaning of text and perform tasks close to humans. Indeed, our results showed that model predictions were driven by the DILI-related words such as hepatic failure, hepatotoxicity and hepatitis in the representative DILI positive sentences. For the representative DILI positive sentences, model predictions were based on the detection of DILI-negative information including chronic hepatitis B/C, even though DILI-related words were also present in the sentence.

Additionally, we acknowledge the following limitations of this study. The dataset size is relatively small, especially for document-level classification results. This is by large due to that DILI is not a common adverse event, with an incidence of approximately 20 cases per 100,000 persons annually (Garcia-Cortes et al., 2020). There are limited number of drugs carrying warnings for DILI. The developed pipeline was evaluated on just a single topic, i.e., liver injury. Thus, it remains to be proven by future research that this framework is indeed extensible to other topics. The pre-trained BERT model was trained on corpuses using general language. Drug labeling, however, uses many domain-specific terms. Further in-domain training of the BERT model might improve the model performance. Also, we did not try other transformers models such as GPT-2 (Radford et al., 2019) and XLNet (Yang et al., 2019) for comparison. The main purpose of this work was to test the applicability of modern language models on regulatory documents, rather than select better models.

## Conclusion

In the current study we demonstrated that AI-based NLP tools performed well in DILI classification of drug labeling documents from two different regulatory agencies, FDA and EMA. The deep learning-based and hybrid deep learning-based models outperformed the keywords-based models and were portable from one agency to the other without a notable decrease in performance. Our results suggest that AI models are able to learn the meaning of text and handle NLP tasks with good accuracy. This proof-of-concept work show that using AI technology to facilitate regulatory activities is a promising approach to modernize and advance regulatory science.

## Data Availability

Publicly available datasets were analyzed in this study. This data can be found here: https://nctr-crs.fda.gov/fdalabel/ui/search.
