# Supplementary material for: BERT-Based Natural Language Processing of Drug Labeling Documents: A Case Study for Classifying Drug-Induced Liver Injury Risk
Source: Front Artif Intell. 2021 Dec 6;4:729834. doi: 10.3389/frai.2021.729834 (PMC8685544; doi:10.3389/frai.2021.729834)
Supplement: Supplementary file 1 [file Table1.DOCX]

**Supplementary Tables and Figures**

Supplementary Table 1. Keywords used for document classifications in this study.

| Keywords used in this study | MedDRA PT for adverse reactions (Demner-Fushman et al., 2018) | MedDRA PT for liver injury (Suzuki et al., 2015) | Keywords for text-mining and human reading (Chen et al., 2011) |
| --- | --- | --- | --- |
| alanine aminotransferase abnormal | Alanine aminotransferase abnormal | Alanine aminotransferase abnormal | ALT/AST |
| alanine aminotransferase elevation | Alanine aminotransferase increased | Alanine aminotransferase increased | ALT/AST |
| alanine aminotransferase increase | Alanine aminotransferase increased | Alanine aminotransferase increased | ALT/AST |
| elevated levels of alanine aminotransferase | Alanine aminotransferase increased | Alanine aminotransferase increased | ALT/AST |
| elevation in alanine aminotransferase | Alanine aminotransferase increased | Alanine aminotransferase increased | ALT/AST |
| elevations alanine aminotransferase | Alanine aminotransferase increased | Alanine aminotransferase increased | ALT/AST |
| elevations in alanine aminotransferase | Alanine aminotransferase increased | Alanine aminotransferase increased | ALT/AST |
| high alanine aminotransferase | Alanine aminotransferase increased | Alanine aminotransferase increased | ALT/AST |
| increase in alanine aminotransferase | Alanine aminotransferase increased | Alanine aminotransferase increased | ALT/AST |
| increased alanine aminotransferase | Alanine aminotransferase increased | Alanine aminotransferase increased | ALT/AST |
| increases in alanine aminotransferase | Alanine aminotransferase increased | Alanine aminotransferase increased | ALT/AST |
| increases in serum alanine aminotransferase | Alanine aminotransferase increased | Alanine aminotransferase increased | ALT/AST |
| serum alanine aminotransferase elevations | Alanine aminotransferase increased | Alanine aminotransferase increased | ALT/AST |
| veno-occlusive |  |  | veno-occlusive disease |
| sgpt increased | Alanine aminotransferase increased | Alanine aminotransferase increased | SGPT/SGOT |
| sgot increased | Aspartate aminotransferase increased | Aspartate aminotransferase increased | SGPT/SGOT |
| aspartate aminotransferase abnormal | Aspartate aminotransferase abnormal | Aspartate aminotransferase abnormal | ALT/AST |
| aspartate aminotransferase elevation | Aspartate aminotransferase increased | Aspartate aminotransferase increased | ALT/AST |
| aspartate aminotransferase increased | Aspartate aminotransferase increased | Aspartate aminotransferase increased | ALT/AST |
| elevation aspartate aminotransferase | Aspartate aminotransferase increased | Aspartate aminotransferase increased | ALT/AST |
| elevations aspartate aminotransferase | Aspartate aminotransferase increased | Aspartate aminotransferase increased | ALT/AST |
| elevations in aspartate aminotransferase | Aspartate aminotransferase increased | Aspartate aminotransferase increased | ALT/AST |
| elevations of aspartate aminotransferase | Aspartate aminotransferase increased | Aspartate aminotransferase increased | ALT/AST |
| increased aspartate aminotransferase | Aspartate aminotransferase increased | Aspartate aminotransferase increased | ALT/AST |
| increases in aspartate aminotransferase | Aspartate aminotransferase increased | Aspartate aminotransferase increased | ALT/AST |
| increases in serum aspartate aminotransferase | Aspartate aminotransferase increased | Aspartate aminotransferase increased | ALT/AST |
| abnormalities of alkaline phosphatase | Blood alkaline phosphatase abnormal | Blood alkaline phosphatase increased | liver enzyme |
| alkaline phosphatase increased | Blood alkaline phosphatase increased | Blood alkaline phosphatase increased | liver enzyme |
| blood alkaline phosphatase increased | Blood alkaline phosphatase increased | Blood alkaline phosphatase increased | liver enzyme |
| elevated alkaline phosphatase | Blood alkaline phosphatase increased | Blood alkaline phosphatase increased | liver enzyme |
| increased alkaline phosphatase | Blood alkaline phosphatase increased | Blood alkaline phosphatase increased | liver enzyme |
| increased blood alkaline phosphatase | Blood alkaline phosphatase increased | Blood alkaline phosphatase increased | liver enzyme |
| increased serum alkaline phosphatase | Blood alkaline phosphatase increased | Blood alkaline phosphatase increased | liver enzyme |
| increases in alkaline phosphatase | Blood alkaline phosphatase increased | Blood alkaline phosphatase increased | liver enzyme |
| elevated liver enzymes | Hepatic enzyme increased | Hepatic enzyme increased | liver enzyme |
| elevation of liver enzymes | Hepatic enzyme increased | Hepatic enzyme increased | liver enzyme |
| elevations in liver enzymes | Hepatic enzyme increased | Hepatic enzyme increased | liver enzyme |
| increased liver enzymes | Hepatic enzyme increased | Hepatic enzyme increased | liver enzyme |
| liver enzyme abnormalities | Hepatic enzyme abnormal | Hepatic enzyme abnormal | liver enzyme |
| liver enzyme elevation | Hepatic enzyme increased | Hepatic enzyme increased | liver enzyme |
| liver enzyme elevations | Hepatic enzyme increased | Hepatic enzyme increased | liver enzyme |
| liver enzyme increases | Hepatic enzyme increased | Hepatic enzyme increased | liver enzyme |
| hyperbilirubinemia | Hyperbilirubinaemia | Hyperbilirubinaemia | hyperbilirubinemia |
| indirect hyperbilirubinemia | Hyperbilirubinaemia | Hyperbilirubinaemia | hyperbilirubinemia |
| unconjugated hyperbilirubinemia | Hyperbilirubinaemia | Hyperbilirubinaemia | hyperbilirubinemia |
| abnormalities of bilirubin | Blood bilirubin abnormal | Blood bilirubin abnormal | liver/hepatic dysfunction |
| abnormalities of total bilirubin | Blood bilirubin abnormal | Blood bilirubin abnormal | liver/hepatic dysfunction |
| bilirubin elevations | Blood bilirubin increased | Blood bilirubin increased | liver/hepatic dysfunction |
| bilirubin increased | Blood bilirubin increased | Blood bilirubin increased | liver/hepatic dysfunction |
| bilirubin total increased | Blood bilirubin increased | Blood bilirubin increased | liver/hepatic dysfunction |
| blood bilirubin increased | Blood bilirubin increased | Blood bilirubin increased | liver/hepatic dysfunction |
| elevated bilirubin | Blood bilirubin increased | Blood bilirubin increased | liver/hepatic dysfunction |
| elevated bilirubin levels | Blood bilirubin increased | Blood bilirubin increased | liver/hepatic dysfunction |
| elevated levels of bilirubin | Blood bilirubin increased | Blood bilirubin increased | liver/hepatic dysfunction |
| elevated serum bilirubin | Blood bilirubin increased | Blood bilirubin increased | liver/hepatic dysfunction |
| elevated total bilirubin | Blood bilirubin increased | Blood bilirubin increased | liver/hepatic dysfunction |
| elevation direct bilirubin | Bilirubin conjugated increased | Bilirubin conjugated increased | liver/hepatic dysfunction |
| elevation in bilirubin | Blood bilirubin increased | Blood bilirubin increased | liver/hepatic dysfunction |
| elevation indirect bilirubin | Blood bilirubin unconjugated increased | Blood bilirubin unconjugated increased | liver/hepatic dysfunction |
| elevation of serum bilirubin | Blood bilirubin increased | Blood bilirubin increased | liver/hepatic dysfunction |
| elevations in bilirubin | Blood bilirubin increased | Blood bilirubin increased | liver/hepatic dysfunction |
| elevations in total bilirubin | Blood bilirubin increased | Blood bilirubin increased | liver/hepatic dysfunction |
| elevations of bilirubin | Blood bilirubin increased | Blood bilirubin increased | liver/hepatic dysfunction |
| elevations of serum bilirubin | Blood bilirubin increased | Blood bilirubin increased | liver/hepatic dysfunction |
| elevations of total bilirubin | Blood bilirubin increased | Blood bilirubin increased | liver/hepatic dysfunction |
| high total bilirubin | Blood bilirubin increased | Blood bilirubin increased | liver/hepatic dysfunction |
| increase in bilirubin levels | Blood bilirubin increased | Blood bilirubin increased | liver/hepatic dysfunction |
| increase in total bilirubin | Blood bilirubin increased | Blood bilirubin increased | liver/hepatic dysfunction |
| increased bilirubin | Blood bilirubin increased | Blood bilirubin increased | liver/hepatic dysfunction |
| increased blood bilirubin | Blood bilirubin increased | Blood bilirubin increased | liver/hepatic dysfunction |
| increased conjugated bilirubin | Bilirubin conjugated increased | Bilirubin conjugated increased | liver/hepatic dysfunction |
| increased serum bilirubin | Blood bilirubin increased | Blood bilirubin increased | liver/hepatic dysfunction |
| increased total bilirubin | Blood bilirubin increased | Blood bilirubin increased | liver/hepatic dysfunction |
| increased unconjugated blood bilirubin | Blood bilirubin unconjugated increased | Blood bilirubin unconjugated increased | liver/hepatic dysfunction |
| increases in bilirubin | Blood bilirubin increased | Blood bilirubin increased | liver/hepatic dysfunction |
| increases in serum bilirubin | Blood bilirubin increased | Blood bilirubin increased | liver/hepatic dysfunction |
| increases in total bilirubin | Blood bilirubin increased | Blood bilirubin increased | liver/hepatic dysfunction |
| increases of bilirubin | Blood bilirubin increased | Blood bilirubin increased | liver/hepatic dysfunction |
| total bilirubin elevation | Blood bilirubin increased | Blood bilirubin increased | liver/hepatic dysfunction |
| total bilirubin elevations | Blood bilirubin increased | Blood bilirubin increased | liver/hepatic dysfunction |
| total bilirubin greater than 2*uln | Blood bilirubin increased | Blood bilirubin increased | liver/hepatic dysfunction |
| total bilirubin greater than 3.0*uln | Blood bilirubin increased | Blood bilirubin increased | liver/hepatic dysfunction |
| total bilirubin greater than or equal to 1.5 x uln | Blood bilirubin increased | Blood bilirubin increased | liver/hepatic dysfunction |
| total bilirubin increased | Blood bilirubin increased | Blood bilirubin increased | liver/hepatic dysfunction |
| total bilirubin two times the uln | Blood bilirubin increased | Blood bilirubin increased | liver/hepatic dysfunction |
| jaundice | Jaundice | Jaundice | jaundice |
| hepatic steatosis | Hepatic steatosis |  | steatosis |
| steatosis | Hepatic steatosis |  | steatosis |
| fatty liver |  |  | fatty liver |
| steatohepatitis | Steatohepatitis |  | steatohepatitis |
| hepatocellular injury | Hepatocellular injury | Hepatocellular injury | liver/hepatic injury |
| hepatocellular liver injury | Hepatocellular injury | Hepatocellular injury | liver/hepatic injury |
| cholestasis | Cholestasis | Cholestasis | cholestasis |
| cholestatic hepatitis |  | Hepatitis cholestatic | cholestatic hepatitis |
| eosinophilic hepatitis |  |  | hepatitis |
| hepatopathy |  |  | hepatopathy |
| hepatomegaly | Hepatomegaly |  | hepatomegaly |
| hepatocyte necrosis | Hepatic necrosis | Hepatic necrosis | liver/hepatic necrosis |
| liver necrosis | Hepatic necrosis | Hepatic necrosis | liver/hepatic necrosis |
| hepatic necrosis | Hepatic necrosis | Hepatic necrosis | liver/hepatic necrosis |
| acute liver failure | Acute hepatic failure | Acute hepatic failure | liver/hepatic failure |
| liver failure | Hepatic failure | Hepatic failure | liver/hepatic failure |
| hepatic failure | Hepatic failure | Hepatic failure | liver/hepatic failure |
| acute hepatic failure | Hepatic failure |  | liver/hepatic failure |
| hepatic abnormalities |  |  | liver/hepatic dysfunction |
| liver dysfunction | Hepatic function abnormal | Hepatic function abnormal | liver/hepatic dysfunction |
| hepatic dysfunction | Hepatic function abnormal | Hepatic function abnormal | liver/hepatic dysfunction |
| liver encephalopathy | Hepatic encephalopathy |  | liver/hepatic encephalopathy |
| hepatic encephalopathy | Hepatic encephalopathy |  | liver/hepatic encephalopathy |
| liver transplantation |  |  | liver/hepatic transplantation |
| hepatic transplantation |  |  | liver/hepatic transplantation |
| hepatotoxicity | Hepatotoxicity | Hepatotoxicity | hepatotoxicity |
| hepatotoxicity adverse events | Hepatotoxicity | Hepatotoxicity | hepatotoxicity |
| liver toxicity | Hepatotoxicity | Hepatotoxicity | hepatotoxicity |
| hepatic toxicity | Hepatotoxicity | Hepatotoxicity | hepatotoxicity |
| drug induced liver injury | Drug-induced liver injury |  | liver/hepatic injury |
| drug-related liver injury | Drug-induced liver injury |  | liver/hepatic injury |
| hepatocellular liver injury | Hepatocellular injury | Hepatocellular injury | liver/hepatic injury |
| liver injury | Liver injury | Liver injury | liver/hepatic injury |
| hepatic injury | Liver injury | Liver injury | liver/hepatic injury |
| liver reaction |  |  | liver/hepatic reaction |
| hepatic reaction |  |  | liver/hepatic reaction |
| abnormalities in liver function tests | Liver function test abnormal | Liver function test abnormal | liver/hepatic dysfunction |
| liver function abnormalities | Hepatic function abnormal | Hepatic function abnormal | liver/hepatic dysfunction |
| liver function test abnormal | Liver function test abnormal | Liver function test abnormal | liver/hepatic dysfunction |
| liver function test abnormalities | Liver function test abnormal | Liver function test abnormal | liver/hepatic dysfunction |
| abnormal liver function | Hepatic function abnormal | Hepatic function abnormal | liver/hepatic dysfunction |
| liver function abnormal | Hepatic function abnormal | Hepatic function abnormal | liver/hepatic dysfunction |
| abnormal hepatic function | Hepatic function abnormal | Hepatic function abnormal | liver/hepatic dysfunction |
| hepatic function abnormal | Hepatic function abnormal | Hepatic function abnormal | liver/hepatic dysfunction |
| abnormalities in hepatic function tests | Liver function test abnormal | Liver function test abnormal | liver/hepatic dysfunction |
| hepatic function abnormalities | Hepatic function abnormal | Hepatic function abnormal | liver/hepatic dysfunction |
| hepatic function test abnormal | Liver function test abnormal | Liver function test abnormal | liver/hepatic dysfunction |
| hepatic function test abnormalities | Liver function test abnormal | Liver function test abnormal | liver/hepatic dysfunction |
| hepatobiliary disorders | Hepatobiliary disease |  | liver/hepatic disorder |
| hepatobiliary laboratory abnormalities | Liver function test abnormal | Liver function test abnormal | liver/hepatic dysfunction |
| aspartate transaminase increased | Aspartate aminotransferase increased | Aspartate aminotransferase increased | ALT/AST |
| elevated hepatic transaminases | Transaminases increased | Transaminases increased | liver enzyme |
| elevated serum transaminase levels | Transaminases increased | Transaminases increased | liver enzyme |
| elevated serum transaminases | Transaminases increased | Transaminases increased | liver enzyme |
| elevated transaminases | Transaminases increased | Transaminases increased | liver enzyme |
| elevation of liver transaminases | Transaminases increased | Transaminases increased | liver enzyme |
| elevations in hepatic transaminases | Transaminases increased | Transaminases increased | liver enzyme |
| elevations in liver transaminases | Transaminases increased | Transaminases increased | liver enzyme |
| elevations in serum transaminases | Transaminases increased | Transaminases increased | liver enzyme |
| elevations in transaminases | Transaminases increased | Transaminases increased | liver enzyme |
| elevations of serum hepatic transaminases | Transaminases increased | Transaminases increased | liver enzyme |
| elevations of transaminases | Transaminases increased | Transaminases increased | liver enzyme |
| hepatic transaminases increased | Transaminases increased | Transaminases increased | liver enzyme |
| increase in transaminase levels | Transaminases increased | Transaminases increased | liver enzyme |
| increased serum transaminases | Transaminases increased | Transaminases increased | liver enzyme |
| increased transaminases | Transaminases increased | Transaminases increased | liver enzyme |
| increases in serum glutamic-oxaloacetic transaminase | Aspartate aminotransferase increased | Aspartate aminotransferase increased | SGPT/SGOT |
| increases in serum glutamic-pyruvic transaminase | Alanine aminotransferase increased | Alanine aminotransferase increased | SGPT/SGOT |
| increases in serum transaminase levels | Transaminases increased | Transaminases increased | liver enzyme |
| increases in serum transaminases | Transaminases increased | Transaminases increased | liver enzyme |
| increases in transaminases | Transaminases increased | Transaminases increased | liver enzyme |
| liver transaminase elevation | Transaminases increased | Transaminases increased | liver enzyme |
| serum transaminase elevations | Transaminases increased | Transaminases increased | liver enzyme |
| transaminase elevation | Transaminases increased | Transaminases increased | liver enzyme |
| transaminase elevations | Transaminases increased | Transaminases increased | liver enzyme |
| transaminase increased | Transaminases increased | Transaminases increased | liver enzyme |
| transaminases abnormal | Transaminases abnormal | Transaminases abnormal | liver enzyme |
| transaminases increased | Transaminases increased | Transaminases increased | liver enzyme |
| worsening transaminase elevations | Transaminases increased | Transaminases increased | liver enzyme |
| abnormalities in alanine transaminase | Alanine aminotransferase abnormal | Alanine aminotransferase abnormal | ALT/AST |
| alanine transaminase increased | Alanine aminotransferase increased | Alanine aminotransferase increased | ALT/AST |
| abnormalities in alt | Alanine aminotransferase abnormal | Alanine aminotransferase abnormal | ALT/AST |
| alt abnormalities | Alanine aminotransferase abnormal | Alanine aminotransferase abnormal | ALT/AST |
| alt elevation | Alanine aminotransferase increased | Alanine aminotransferase increased | ALT/AST |
| alt elevations | Alanine aminotransferase increased | Alanine aminotransferase increased | ALT/AST |
| alt increase | Alanine aminotransferase increased | Alanine aminotransferase increased | ALT/AST |
| alt increased | Alanine aminotransferase increased | Alanine aminotransferase increased | ALT/AST |
| alt increases | Alanine aminotransferase increased | Alanine aminotransferase increased | ALT/AST |
| elevated alt | Alanine aminotransferase increased | Alanine aminotransferase increased | ALT/AST |
| elevated serum levels alt | Alanine aminotransferase increased | Alanine aminotransferase increased | ALT/AST |
| elevation alt | Alanine aminotransferase increased | Alanine aminotransferase increased | ALT/AST |
| elevation in alt | Alanine aminotransferase increased | Alanine aminotransferase increased | ALT/AST |
| elevations alt | Alanine aminotransferase increased | Alanine aminotransferase increased | ALT/AST |
| elevations in alt | Alanine aminotransferase increased | Alanine aminotransferase increased | ALT/AST |
| high alt | Alanine aminotransferase increased | Alanine aminotransferase increased | ALT/AST |
| increase in alt | Alanine aminotransferase increased | Alanine aminotransferase increased | ALT/AST |
| increased alt | Alanine aminotransferase increased | Alanine aminotransferase increased | ALT/AST |
| increases in alt | Alanine aminotransferase increased | Alanine aminotransferase increased | ALT/AST |
| serum alt increased | Alanine aminotransferase increased | Alanine aminotransferase increased | ALT/AST |
| elevated ast | Aspartate aminotransferase increased | Aspartate aminotransferase increased | ALT/AST |
| elevation ast | Aspartate aminotransferase increased | Aspartate aminotransferase increased | ALT/AST |
| elevation in ast | Aspartate aminotransferase increased | Aspartate aminotransferase increased | ALT/AST |
| elevations ast | Aspartate aminotransferase increased | Aspartate aminotransferase increased | ALT/AST |
| elevations in ast | Aspartate aminotransferase increased | Aspartate aminotransferase increased | ALT/AST |
| elevations of ast | Aspartate aminotransferase increased | Aspartate aminotransferase increased | ALT/AST |
| high ast | Aspartate aminotransferase increased | Aspartate aminotransferase increased | ALT/AST |
| increase in ast | Aspartate aminotransferase increased | Aspartate aminotransferase increased | ALT/AST |
| increased ast | Aspartate aminotransferase increased | Aspartate aminotransferase increased | ALT/AST |
| increases in ast | Aspartate aminotransferase increased | Aspartate aminotransferase increased | ALT/AST |
| increases in serum ast | Aspartate aminotransferase increased | Aspartate aminotransferase increased | ALT/AST |
| increases of ast | Aspartate aminotransferase increased | Aspartate aminotransferase increased | ALT/AST |
| alp increased | Blood alkaline phosphatase increased | Blood alkaline phosphatase increased | ALT/AST |
| increased alp | Blood alkaline phosphatase increased | Blood alkaline phosphatase increased | ALT/AST |
| elevations in hepatic enzymes | Hepatic enzyme increased | Hepatic enzyme increased | liver enzyme |
| hepatic enzyme elevation | Hepatic enzyme increased | Hepatic enzyme increased | liver enzyme |
| hepatic enzyme elevations | Hepatic enzyme increased | Hepatic enzyme increased | liver enzyme |
| hepatic enzyme increased | Hepatic enzyme increased | Hepatic enzyme increased | liver enzyme |
| increased hepatic enzymes | Hepatic enzyme increased | Hepatic enzyme increased | liver enzyme |
| autoimmune hepatitis | Autoimmune hepatitis |  | hepatitis |
| cytolytic hepatitis | Hepatitis | Cytolytic hepatitis | hepatitis |
| fulminant hepatitis | Hepatitis fulminant | Hepatitis fulminant | hepatitis |
| hepatitis | Hepatitis | Cytolytic hepatitis | hepatitis |
| immune-mediated hepatitis | Autoimmune hepatitis |  | hepatitis |
| steatohepatitis | Steatohepatitis |  | hepatitis |
| toxic hepatitis | Hepatitis toxic | Hepatitis toxic | hepatitis |
| acute hepatitis |  | Hepatitis acute | hepatitis |
| cholestatic jaundice |  | Jaundice cholestatic | jaundice |
| hepatocellular jaundice |  | Jaundice hepatocellular | jaundice |
| yellow skin |  | Yellow skin | jaundice |
| urine bilirubin increased |  | Urine bilirubin increased |  |
| bilirubin urine |  | Bilirubin urine |  |
| subacute hepatic failure |  | Subacute hepatic failure | liver/hepatic failure |
| mitochondrial hepatopathy |  | Mitochondrial hepatopathy | hepatopathy |
| cholestatic pruritus |  | Cholestatic pruritus |  |
| total bile acids increased |  | Total bile acids increased |  |
| mixed liver injury |  | Mixed liver injury | liver/hepatic injury |
| cholestatic liver injury |  | Cholestatic liver injury | liver/hepatic injury |

Supplementary Table 2. Liver-related context defined by general string patterns matching.

|  | String patterns | Representative terms/phrases matched to the string patterns |
| --- | --- | --- |
| Positive selection | AST | AST increase, AST elevation, etc. |
|  | ALT | ALT increase, ALT elevation, etc. |
|  | ALP | ALP increase, ALP elevation, etc. |
|  | transaminase | increased transaminases, elevations in transaminases, etc. |
|  | aminotransferase | increased aminotransferases, elevations in aminotransferases, etc. |
|  | alkaline phosphatase | increased alkaline phosphatase, elevations in alkaline phosphatase, etc. |
|  | bilirubin | increased bilirubin levels, hyperbilirubinemia, etc. |
|  | liver | liver functions, liver enzymes, liver impairment, liver tests, etc. |
|  | hepatic | hepatic functions, hepatic enzymes, hepatic impairment, hepatic failure, etc. |
|  | hepato | hepatotoxicity, hepatocellular, hepatocyte, hepatobiliary, hepatomegaly, etc. |
|  | hepatitis | hepatitis B, cholestatic hepatitis, steatotic hepatitis, autoimmune hepatitis, etc. |
|  | jaundice | jaundice |
|  | cholestasis | cholestasis |
|  | cholestatic | cholestatic liver injury, cholestatic hepatitis, etc. |
|  | steatosis | steatosis |
|  | steatotic | steatotic hepatitis, steatotic liver injury, etc. |
|  | veno-occlusive | hepatic veno-occlusive disease |
|  | liver encephalopathy | liver encephalopathy |
|  | hepatic encephalopathy | hepatic encephalopathy |
| Negative selection | mice | sentences from animal studies |
|  | mouse |  |
|  | rat |  |
|  | dog |  |
|  | animal |  |

Supplementary Table 3. Hyperparameters used for training the BERT-NER-based context classification module.

|  | model training |
| --- | --- |
| number of tokens | 128 |
| batch size | 32 |
| optimizer | AdamW |
| learning rate | 2E-05 |
| epochs | 4 |

Supplementary Table 4. Hyperparameters used for training the BERT-based sentence classification module.

|  | model training |
| --- | --- |
| number of tokens | 128 |
| batch size | 16 |
| optimizer | AdamW |
| learning rate | 2E-05 |
| epochs | 3 |

Supplementary Table 5. Categories of false positives produced by keyword-based document classification but not by the FDA model.

| Reasons for False Positive | Count | Percentage |
| --- | --- | --- |
| special population - patients with hepatic impairment | 10 | 40% |
| hypersensitivity | 6 | 24% |
| complications of other disorders | 3 | 12% |
| indication | 2 | 8% |
| baseline assessment of liver function | 1 | 4% |
| concomitant medication | 1 | 4% |
| immunosuppressants | 1 | 4% |
| reactivation of virus | 1 | 4% |

Supplementary Table 6. Model evaluation using EMA test documents and validation using cross-agency data

| Model evaluation using EMA test documents | | | |
| --- | --- | --- | --- |
| Document classification models | Matthews correlation coefficient | Recall | Precision |
| AI model | 0.73 | 0.79 | 0.82 |
| Hybrid AI model | 0.89 | 1.00 | 0.85 |
| Model validation using cross-agency data (FDA documents) | | | |
| Document classification models | Matthews correlation coefficient | Recall | Precision |
| AI model | 0.79 | 0.82 | 0.86 |
| Hybrid AI model | 0.87 | 0.96 | 0.85 |


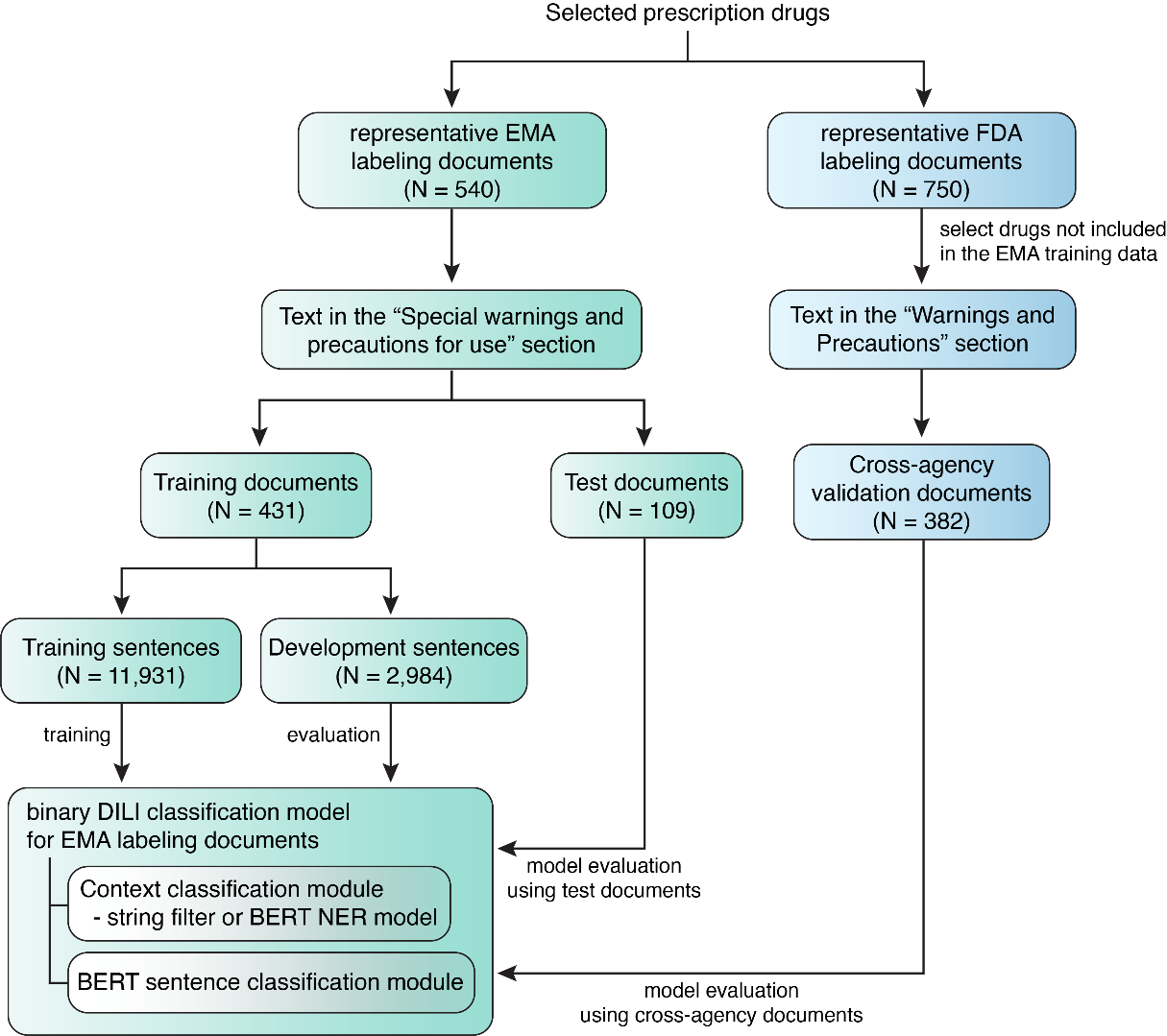


**Supplementary Figure 1. Quorum flowchart describes the study design** **of model training and evaluation using FDA labeling documents and model validation using EMA labeling documents.**


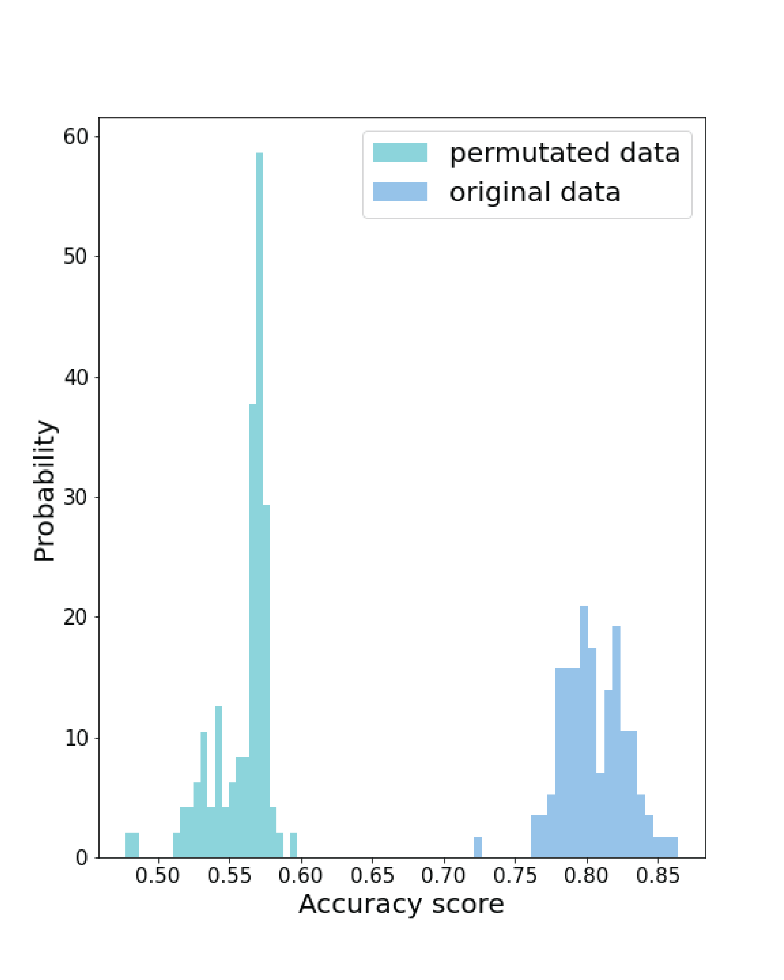


**Supplementary Figure 2. Permutation tests of the AI models trained on FDA or EMA data.** (A) distribution of accuracy scores obtained from models trained on the permutated FDA datasets (N = 100) or cross-validations using the original datasets (N = 100). (B) distribution of accuracy scores obtained from models trained on the permutated EMA datasets (N = 100) or cross-validations using the original datasets (N = 100).
